# Supplementary material for: Content-rich biological network constructed by mining PubMed abstracts
Source: BMC Bioinformatics. 2004 Oct 8;5:147. doi: 10.1186/1471-2105-5-147 (PMC528731; doi:10.1186/1471-2105-5-147)
Supplement: Additional File 2 — The original results of the above study (non-essential files are deleted to keep the file size under the limit set by BMC bioinformatics). [file 1471-2105-5-147-S2.bz2 › chilibotAdditionalFile2/dip20/494ID10187774E10657/html/TGFBR1_TGFB3.html]

 


 **TGFBR1** and **TGFB3** 
  
Found 25 abstracts in PubMed, retrieved 20.  
 

 What does Google say? 
 PDF only 
| .edu only 

---

**Interactive relationship** (e.g. stimulation, inhibition, etc)

**Non-interactive relationship** (e.g. studied together, co-existance, homology, etc.)

- In this study, we analyze the expression of TGF beta1, TGF beta2, and TGF beta3  [ **TGFB3** ]  and their receptors type I Tbeta RI ALK5  [ **TGFBR1** ] , type II Tbeta RII, and type III Tbeta RIII in NP.  Ref: 9445116 Ann Surg, 1998
- We therefore studied the expression of UPA, UPAR, IGF2R, ALK5  [ **TGFBR1** ] , TGFBR2, TGFBR3, ENG, ALK1, TGFB1, TGFB2, and  **TGFB3**  in a series of 14 pancreatic carcinoma cell lines.  Ref: 11408925 Int J Oncol, 2001
- METHODS AND RESULTS The spatial relationships between TGF beta1, TGF beta3  [ **TGFB3** ] , ALK 5  [ **TGFBR1** ] , and TbetaR II expression were compared in aortic segments from 21 subjects.  Ref: 10359732 Circulation, 1999
- Tranilast attenuated in a dose dependent and reversible manner the injury induced increases in mRNA levels encoding TGF beta1, TGF beta3  [ **TGFB3** ] , two type I TGF beta receptors ALK 5  [ **TGFBR1** ]  and ALK 2, and the type II receptor TbetaRII.  Ref: 9622270 Atherosclerosis, 1998
- In this study we have investigated the expression of the precursor forms of TGF beta1, TGF beta2, TGF beta3  [ **TGFB3** ] , as well as TGF beta type I receptor  [ **TGFBR1** ]  TGF betaRI and TGF beta type II receptor TGF betaRII in the synovial tissue of arthritic rats during the course of the disease.  Ref: 9010265 Clin Exp Immunol, 1997
- Here, we characterize the correlative expression pattern of the interdependent high affinity receptor proteins for TGF beta1 and TGF beta3  [ **TGFB3** ] , TGF beta receptor type I  [ **TGFBR1** ]  TGF betaRI and TGF beta receptor type II TGF betaRII, during hair follicle development and cycling in C57BL 6 mice.  Ref: 9326384 J Invest Dermatol, 1997
- Messenger RNAs encoding TGF beta3  [ **TGFB3** ] , ALK 5  [ **TGFBR1** ]  and ALK 2 were all attenuated by between 70 and 74% all P <.  Ref: 9622270 Atherosclerosis, 1998
- Immunohistochemical analysis demonstrated intense TGF beta1, TGF beta2, TGF beta3  [ **TGFB3** ] , Tbeta RI ALK5  [ **TGFBR1** ] , and Tbeta RII immunoreactivity in the remaining acinar and ductal cells in most NP samples.  Ref: 9445116 Ann Surg, 1998
- There were also associated increases in TGF beta 1, TGF beta 3  [ **TGFB3** ] , ALK 5  [ **TGFBR1** ] , and T beta RII immunoreactive peptide levels.  Ref: 9409216 Arterioscler Thromb Vasc Biol, 1997
- Furthermore, competition based quantitative reverse transcription PCR using synthetic internal standards revealed that leiomyomata express a significantly higher number copies per cell of TGF beta type II receptor mRNA, followed by TGF beta 1, TGF beta type I receptor  [ **TGFBR1** ] , TGF beta 2, and TGF beta 3  [ **TGFB3** ]  P <.  Ref: 8784073 J Clin Endocrinol Metab, 1996
- Boston minipigs, treated with tranilast or vehicle, were subjected to endoluminal stenting, and the expression of TGF beta1 and TGF beta3  [ **TGFB3** ] , the expression of their signaling receptors ALK 5  [ **TGFBR1** ]  and TbetaR II, leukocyte numbers around the stent struts, and neointima development were assessed over 28 days.  Ref: 12067902 Arterioscler Thromb Vasc Biol, 2002
- Although neither TGF beta2 nor TGF beta3  [ **TGFB3** ]  expression was affected by hypoxia, the TGF beta type I receptor  [ **TGFBR1** ]  was substantially upregulated within 6 h.  Ref: 11421617 J Surg Res, 2001
- METHODS The expression of TGF beta1, TGF beta2, TGF beta3  [ **TGFB3** ] , Tbeta RI ALK5  [ **TGFBR1** ] , Tbeta RII, Tbeta RIII, and collagen type I mRNA was analyzed by Northern blot analysis.  Ref: 9445116 Ann Surg, 1998
- In addition, immunohistochemical analysis using polyclonal antibodies was performed to detect TGF beta1, TGF beta2, TGF beta3  [ **TGFB3** ] , Tbeta RI ALK5  [ **TGFBR1** ] , and Tbeta RII.  Ref: 9445116 Ann Surg, 1998
